# Photostabilizing Efficiency of PVC in the Presence of Schiff Bases as Photostabilizers

**DOI:** 10.3390/molecules201119665

**Published:** 2015-11-04

**Authors:** Emad Yousif, Ahmed A. Al-Amiery, Abdulhadi Kadihum, Abdul Amir H. Kadhum, Abu Bakar Mohamad

**Affiliations:** 1 Department of Chemistry, College of Science, Al-Nahrain University, Baghdad 10072, Iraq; emad_yousif@hotmail.com; 2 Environmental Research Center, University of Technology (UOT), Baghdad 10001, Iraq; 3 Department of Chemical & Process Engineering, University Kebangsaan Malaysia (UKM), Bangi, Selangor 43000, Malaysia; amir@eng.ukm.my (A.A.H.K.); drab@eng.ukm.my (A.B.M.); 4 Department of Laser and Optoelectronics, University of Technology (UOT), Baghdad 10001, Iraq; abdulhadikadhim5@gmail.com; 5 Fuel Cell Institute, University Kebangsaan Malaysia (UKM), Bangi, Selangor 43000, Malaysia

**Keywords:** photochemistry, polyvinyl chloride (PVC), photostabilizer, UV absorber, UV-Vis spectroscopy

## Abstract

The photostabilization of polyvinyl chloride (PVC) films by Schiff bases was investigated. Polyvinyl chloride films containing 0.5 wt % Schiff bases were produced using the same casting method as that used for additive-free PVC films from tetrahydrofuran (THF) solvent. The photostabilization activities of these compounds were determined by monitoring the carbonyl, polyene and hydroxyl indices with irradiation time. The changes in viscosity average molecular weight of PVC with irradiation time were also monitored using THF as a solvent. The quantum yield of chain scission (Φ_cs_) for the studied complexes in PVC was estimated to range between 4.72 and 8.99 × 10^−8^. According to the experimental results, several mechanisms were suggested, depending on the structure of the additive. Ultra violet (UV) absorption, peroxide decomposition and radical scavenging were suggested as the photostabilizing mechanisms.

## 1. Introduction

Polyvinyl chloride is the third-most widely produced synthetic plastic polymer after polyethylene and polypropylene and is widely used in several industries, including architecture, electronic, chemical engineering, packaging, and transportation [1,2,3,4]. However, the low photostability of PVC leads to hydrogen chloride loss, discoloration, and serious corrosion phenomena, accompanied by changes in the physical and chemical properties of PVC. The low cost and good performance of polyvinyl chloride products have increased the utilization of this polymer in construction, mainly in exterior applications, such as window profiles, cladding structures and siding [5]. However, user acceptance of the PVC products for outdoor construction applications will depend on their ability to resist photo-degradation over long periods of sunlight exposure [6]. In addition, it is important to perform efficient accelerated experiments and to investigate the effects of degradation factors of polyvinyl chloride under the required conditions of this application. Polymer materials have found a number of important applications in dye-sensitized solar cells (DSSCs) as luminescent and protective coatings [7], templates for designing new nanostructured TiO-electrodes [8], stable electrolytes [9], conductive plastic substrates [10], counter electrodes [11] and other components of solar cells [12]. Over the last 40 years, the versatility of organic chemistry has allowed significant progress in achieving control over the solid-state properties of functional organic molecules, with the attention focused on covalent bonding and on the tailoring of “intramolecular functionality”. Control at the intermolecular level is more elusive but remains crucial for manipulating and optimizing the relevant properties of the functional materials, such as charge transport or luminescence. Threaded molecular wires fabricated with conjugated polymer-based polyrotaxanes offer an example of a “bottom-up” approach to electroluminescent nanostructures incorporating supramolecular design concepts [13,14]. An alternative to adding photostabilizers at high concentrations to the imaging buffer relies on the direct linkage to the fluorophore. However, the working principles of this approach are not yet fully understood [15]. In the continuation of previous studies [16,17,18,19,20,21,22,23], we focused on the photostabilization of PVC using 1,3,4-thiadiazole compounds. To our knowledge, there has been no attempt to investigate the photostabilization of PVC films by Schiff bases containing four 1,3,4-thiadiazole rings; therefore, in this study, we report the design of the Schiff bases and the study of their use as a photostabilizing reagents.

## 2. Results and Discussion

**Figure 1:**
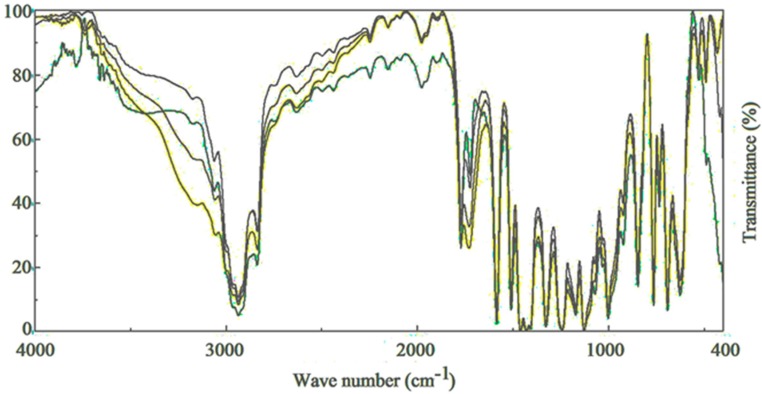
Change in IR spectrum of PVC film (30 µm) in the presence of the III compound (numbers in the spectra are the irradiation times in hours).

**Scheme 1:**
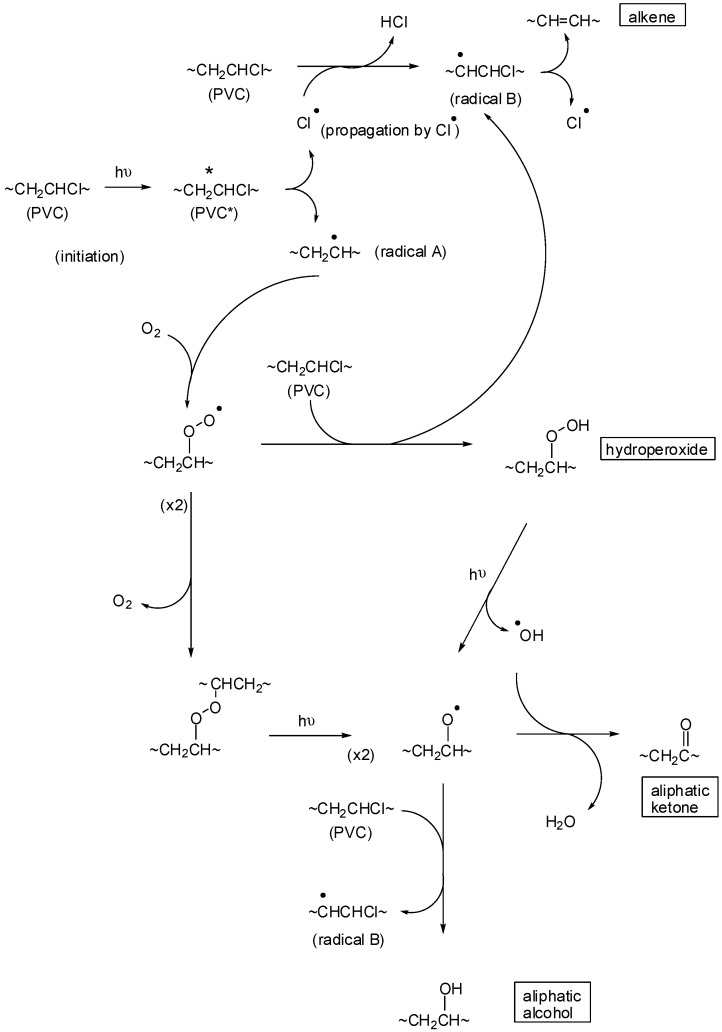
Photo-oxidation reaction scheme of PVC [28]. Where “*” represent excited state and “•” represent free radical.

Schiff bases (I, II, III, IV and V) were used as additives for the photostabilization of PVC films. Previous studies showed that the most effective concentration of additives was 0.5% by weight [24]. Therefore, Schiff bases (I, II, III, IV and V) were used at a concentration of 0.5% by weight for the photostabilization of PVC polymeric films. Exposing the PVC films to light over various radiation times leads to clear changes in their Fourier transform infrared spectroscopy (FTIR) spectra (Figure 1). The two absorption bands appearing at 1770 and 1724 cm^−1^ were attributed to the formation of carbonyl groups. The bands appearing at 1631 and 3400 cm^−1^ were attributed to the formation of a C=C bond conjugated to a carbonyl group, and these absorption bands are in agreement with a recently published study [25]. The absorption bands resonated at 1785 and 1745 cm^−1^ for the carbonyl groups in which the main products were from the photo-oxidation of PVC. The indices for the carbonyl (I_CO_), polyene (I_PO_) and hydroxyl (I_OH_) groups were monitored along with the irradiation time using FTIR spectrophotometry to study the activities of Schiff bases (I, II, III, IV and V) as additives for PVC film photostabilization [26]. The 2N-salicylidene-5-(substituted)-1,3,4-thiadiazole compounds were used as additives for the photo-stabilization of PVC films. To study the photochemical activity of these additives for the photo-stabilization of PVC films, the carbonyl and polyene indices were monitored with irradiation time using IR spectrophotometry (as shown in Scheme 1). The irradiation of PVC films with UV light of wavelength λ = 313 nm led to a clear change in the FT-IR spectrum [27], as shown in Figure 1. The appearance of bands at 1772 cm^−1^ and 1724 cm^−1^ were attributed to the formation of carbonyl groups related to chloro ketone and to aliphatic ketone, respectively.

**Figure 2:**
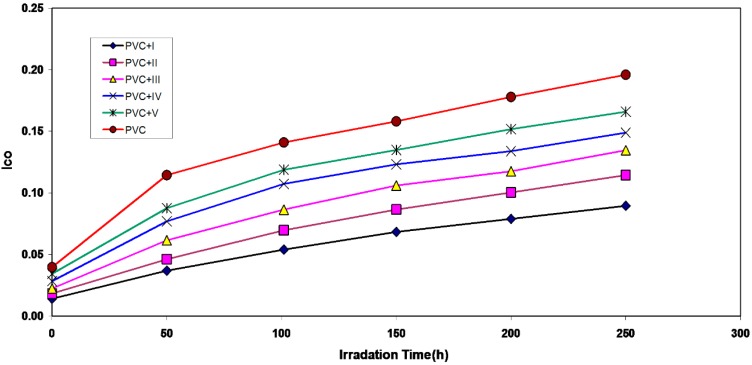
The relationship between the carbonyl index and irradiation time for PVC films (30-μm thickness) containing different additives. The concentration of additives is fixed at 0.5% by weight.

**Figure 3:**
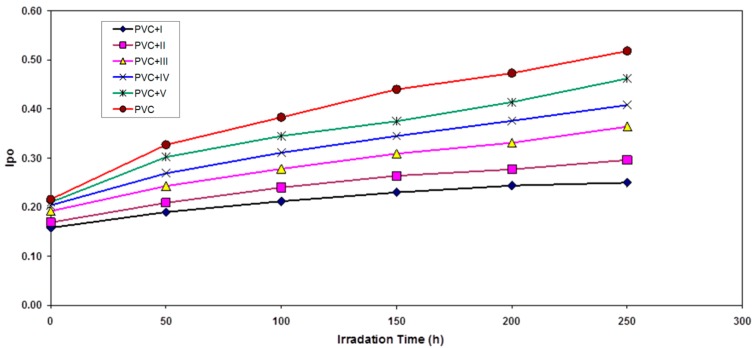
The relationship between the polyene index and irradiation time for PVC films (30-μm thickness) containing different additives. The concentration of additives is fixed at 0.5% by weight.

Carbonyls, hydroxyls and polyenes were utilized to follow the degradation of polymers during irradiation. This absorption was calculated as carbonyl index (I_co_), polyene index (I_PO_) and hydroxyl index (I_OH_). It is reasonable to assume that the growth of the carbonyl index is a measure of the extent of degradation. However, in Figure 2, the (I_co_) of I, II, III, IV and V showed a lower growth rate with irradiation time with respect to the PVC control film without additives. The parallel increase of the carbonyl index together with the time of irradiation was lower than polyvinyl chloride control, as in Figure 2, and it was proper to infer that these additives will be considered as photostabilizers of polyvinyl chloride. Since an efficient photostabilizer shows a longer induction period, therefore, the V is considered as the most active photostabilizer, followed by IV, III, II and I, which is the less active. Just like carbonyl, polyene compounds are also produced during photo-degradation of PVC. Therefore, the polyene index (I_PO_) could also be monitored with irradiation time in the presence and absence of these additives. Results are shown in Figure 3.

**Figure 4:**
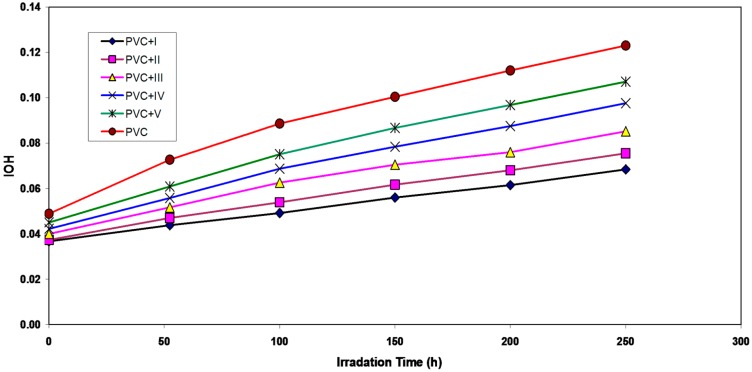
The relationship between the hydroxyl index and irradiation time for PVC films (30-μm thickness) containing different additives. The concentration of additives is fixed at 0.5% by weight.

Hydroxyl species were produced during photo-degradation of PVC. Therefore, the hydroxyl index (I_OH_) was monitored with irradiation time for PVC and with additives. From Figure 4, the V, IV, III, II and I showed lower growth rates of the hydroxyl index with irradiation time compared to pure PVC.

### 2.1. Variation of PVC Molecular Weight during Photolysis in the Presence of 2,5-Dimercapto-1,3,4-Thiadiazole Compounds

**Figure 5:**
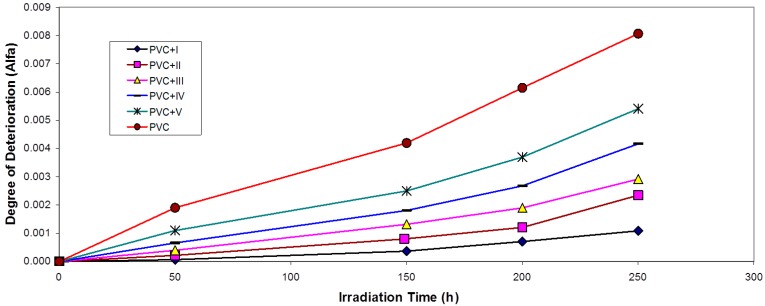
Changes in the viscosity average molecular weight ${\text{(}\bar{\text{M}}}_{\text{v}}\text{)}$ during irradiation of PVC films (30-μm) (control) and with 0.5 wt % of additives.

Analysis of the relative changes in viscosity average molecular weight ${\text{(}\bar{\text{M}}}_{\text{v}}\text{)}$ has been shown to provide a versatile test for random chain scission. Figure 5 shows the plot of $\bar{\text{M}}\text{v}$ *vs.* irradiation time for PVC film with and without 0.5% (wt/wt) of the selected additives, with absorbed light intensity of 1.052 × 10^−8^ Ein·Dm^−3^·S^−1^. $\bar{\text{M}}\text{v}$ is measured using Equation (4) with THF as the solvent at 25 °C.

**Figure 6:**
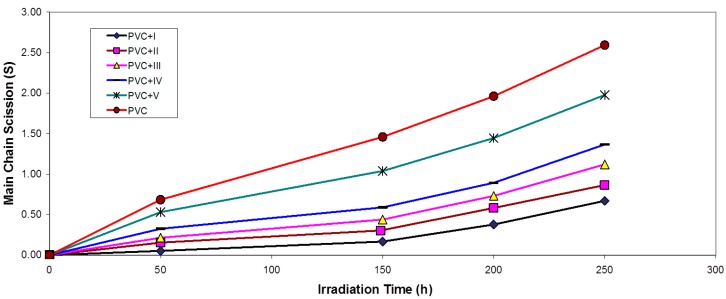
Changes in the main chain scission (S) during irradiation of PVC films (30 μm) (control) and with 0.5 wt % of additives.

It is worth mentioning that traces of the PVC films with additives are not soluble in THF, indicating that cross-linking or branching in the PVC chain does occur during the course of photolysis [29,30]. For better support of this view, the number of average chain scission (average number cut per single chain) (S) [11] was calculated using Equation (1):

(1) $$\text{S}=\frac{{\bar{\text{M}}}_{\text{v,o}}}{{\bar{\text{M}}}_{\text{v,t}}}-1$$

where ${\bar{\text{M}}}_{\text{v,o}}$ and ${\bar{\text{M}}}_{\text{v,t}}$ are viscosity average molecular weight at initial (o) and t irradiation time, respectively. The plot of S *vs.* time is shown in Figure 6. The curve indicates an increase in the degree of branching, such as that might arise from cross-linking occurrence. It is observed that insoluble material was formed during irradiation, which provided additional evidence to the idea that cross-linking occurs [28].

For randomly-distributed weak bond links, which break rapidly in the initial stages of photo-degradation, the degree of deterioration α is given as Equation (2):

(2) $$\alpha =\frac{ms}{{\bar{M}}_{v}}$$

where *m* is the initial molecular weight.

**Figure 7:**
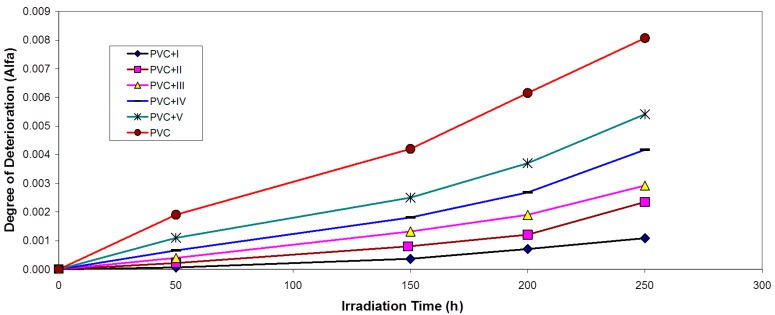
Changes in the degree of deterioration (α) during irradiation of PVC films (30 μm) (control) and with 0.5 wt % of additives.

The plot of α as a function of irradiation time is shown in Figure 7.

The values of α of the irradiated samples are higher when additives are absent and lower in the presence of additives compared with the corresponding values of the additive-free PVC. In the initial stages of PVC photo-degradation, the value of α increases rapidly with time, thus indicating a random breaking of bonds in the polymer chain. Another method of degradation reaction characterization is the measurement of the quantum yield of the chain scission (Φ_cs_). Φ_cs_ values for the complexes are shown in Table 1.

**Table 1:** Quantum yield (Φ_cs_) for the chain scission for PVC film (30 μm) thickness with and without 0.5% (wt/wt) additive after a 250-h irradiation time.

Additive (0.5 wt %)	Quantum Yield of Main Chain Scission (Φ_cs_)
PVC + I	4.72 × 10^−8^
PVC + II	5.24 × 10^−8^
PVC + III	6.64 × 10^−8^
PVC + IV	7.55 × 10^−8^
PVC + V	8.96 × 10^−8^
PVC (pure)	8.54 × 10^−8^

The Φ_cs_ values for PVC films in the presence of additive are lower than that of additive-free PVC (control), which increase in the order:

$$\frac{\text{I},\text{ II},\text{ III},\text{ IV},\text{ V and PVC}}{\to }$$

The explanation for the low values of Φ_cs_ is that in the PVC macromolecule, the energy is absorbed at one site, and then the electronic excitation is distributed over many bonds so that the probability of a single bond breaking is small or the absorbed energy is dissipated by non-reactive processes [31]. It is well established that the quantum yield (Φ_cs_) increases with increasing temperature [32,33] around the glass transition temperature, T_g_, of the amorphous polymer and around the melting temperature of crystalline polymers. In the present study, the photolysis of PVC film is performed at 35–45 °C, which is well below the glass transition temperature (T_g_ of PVC = 80 °C). Therefore, the Φ_cs_ dependency on temperature is not expected to be observed.

### 2.2. Suggested Mechanisms of Photostabilization of PVC by 2,5-Dimercapto-1,3,4-thiadiazole Compounds

**Scheme 2:**
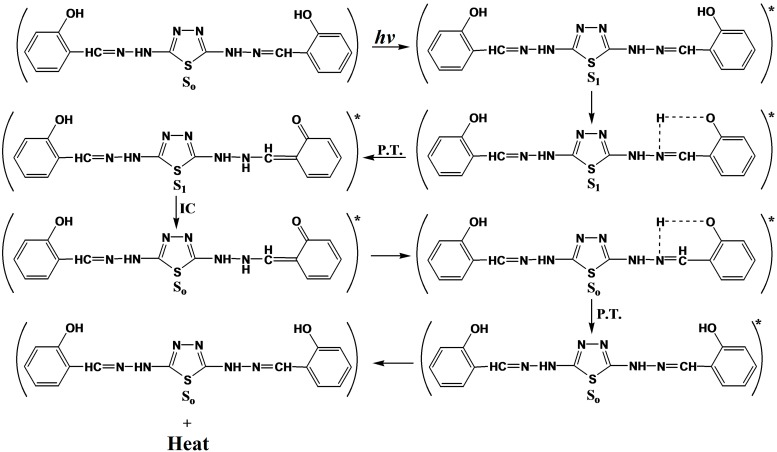
The suggested mechanism of the photostabilization of 2,5-di(arylhydrazones)-1,3,4-thiadiazole compounds through absorption of UV light and dissipation of light energy as heat. Where “*” represent excited state and “•” represent free radical.

**Scheme 3:**
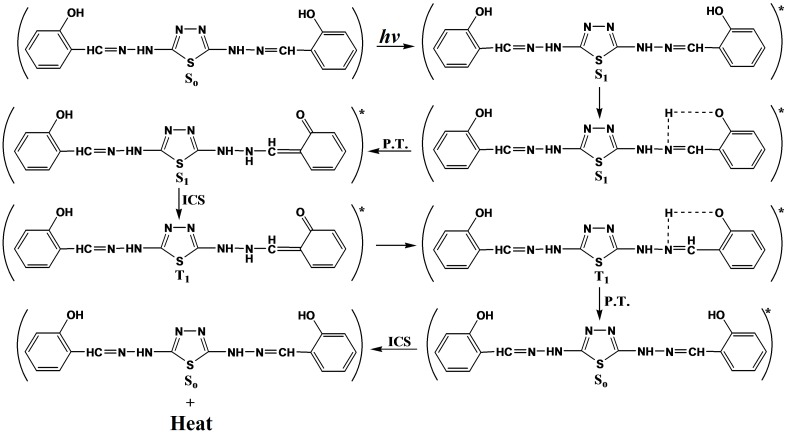
The suggested mechanism of the photostabilization of PVC by 2,5-di(arylhydrazones)-1,3,4-thiadiazole compounds through absorption of UV light and dissipation of light energy as heat. Where “*” represent excited state and “•” represent free radical.

Through the overall results obtained, the efficiency of metal thiadiazole-derived Schiff base complexes as photostabilizers for PVC films can be arranged according to the change in the carbonyl and polyene concentration as a reference for the comparison, as shown in Figure 1, Figure 2, Figure 3, Figure 4, Figure 5, Figure 6 and Figure 7. Schiff bases stabilize PVC by different mechanisms, such as acting as UV absorbers, screeners or radical scavengers. These stabilizers provide very good long-term stability and typically act via these mechanisms. The most probable mechanism involved in a photostabilization is the change in energy of absorbed photons in the intramolecular proton transfer. This reaction may occur via two cycles, as proposed in Scheme 2 and Scheme 3. The first cycle occurs through an intersystem crossing (ICS) process to the excited triplet state, while the second occurs through an internal conversion (IC) process to the ground state. The stabilization of PVC films could occur due to the direct absorption of UV radiation by the Schiff bases (I, II, III, IV and V) in which energy can be dissipated as heat (Scheme 2). Similarly, it is believed that the thiadiazole ring itself could stabilize PVC through the direct absorption effect.

**Scheme 4:**
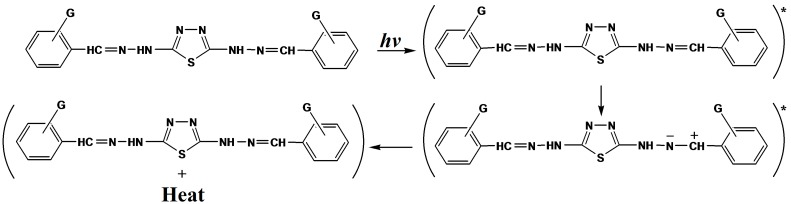
The suggested mechanism of the photostabilization of 2,5-di(aryl hydrazones)-1,3,4-thiadiazole compounds through absorption of UV light and dissipation of light energy as heat. Where “*” represent excited state and “•” represent free radical.

Another mechanism explaining the use of this compound as a photostabilizer is by charge separated species, which could be formed from the excited state. Such a structure would allow dissipation of energy through rotation on increased vibration about the central bond [34], as shown in Scheme 4.

**Scheme 5:**
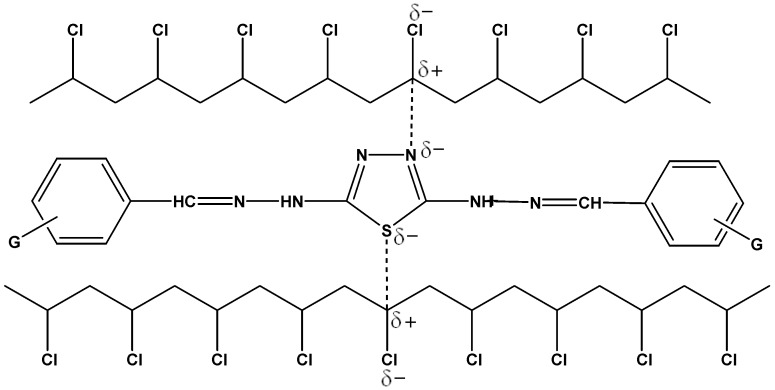
The suggested mechanism of the photostabilization of 2,5-di(arylhydrazones)-1,3,4-thiadiazole compounds through the interaction between PVC and Schiff base compounds.

The interaction between the PVC polymeric chain Schiff base additives has been suggested to be dependent on the coordination between the heteroatoms within Schiff bases, in particular the nitrogen of imine bond, and the polarized atoms of carbon-chlorine bonds within the PVC polymeric chains. It is believed that strong interactions between PVC polymeric chains and Schiff bases could lead to effective energy transfer. Therefore, the attraction between PVC chains and Schiff bases (I, II, III, IV, V), as a result of the polarities of oxygen atoms within the thiadiazole ring, the nitrogen of the imine bonds and the PVC carbon-chlorine bonds, can stabilize the polymeric materials through dissipation of the energy from the PVC excited state by energy transfer, as suggested in Scheme 5. However, there is no experimental evidence to support such a speculation.

The 1,3,4-thiadiazole ring has two different atoms of different electronegativity, such as nitrogen and sulfur. The polarity of this ring explains the attraction between the stabilizer and PVC. This mechanism can lead to the conclusion that crosslinking could take place upon UV irradiation, which may be correct for all compounds (Scheme 5).

**Scheme 6:**
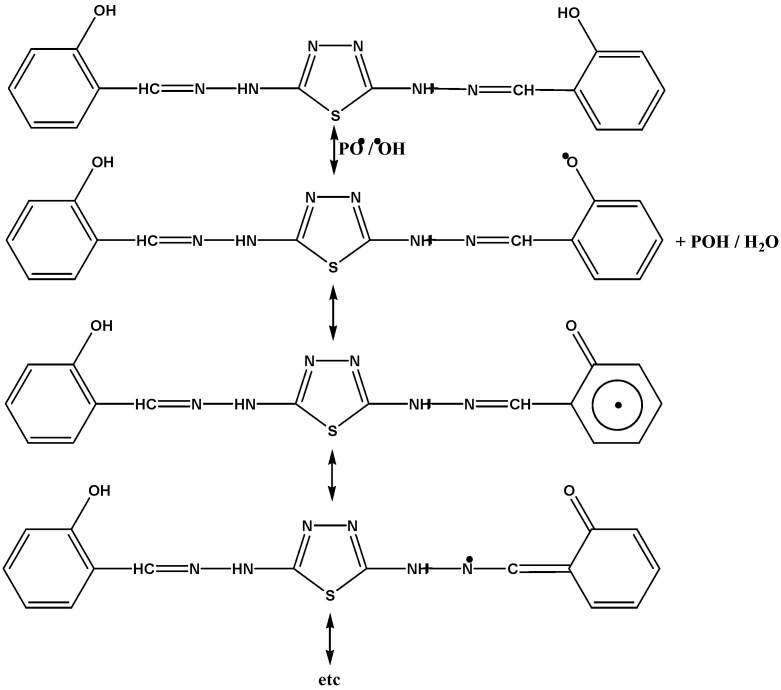
The suggested mechanism of the photostabilization of PVC by the 2,5-di(2-hydroxyl phenyl hydrazone)-1,3,4-thiazole compound as a radical scavenger.

The hydroxyl group of the additive might act as a radical scavenger for the photostabilization process. Therefore, these Schiff bases, besides acting as UV absorbers, may also act as radical scavenger additives, as shown in Scheme 6.

**Scheme 7:**
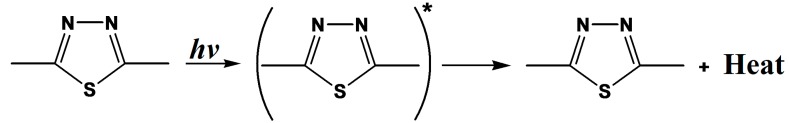
Suggested mechanism of the photostabilization of 1,3,4-thiadiazole as a UV absorber. Where “*” represent excited state and “•” represent free radical.

The rings of 1,3,4-thiadiazole play a role in the mechanism of the stabilizing process by acting as UV absorbers [35]. The UV light absorption by these additives containing 1,3,4-thiadiazole dissipates the UV energy to harmless heat energy (Scheme 7). Furthermore, this ring plays a role in resonating structure conjugation of the radical in peroxide decomposition, as shown in Scheme 7, which explains its function as a photostabilizer.

## 3. Experimental Section

### 3.1. Materials

**Scheme 8:**
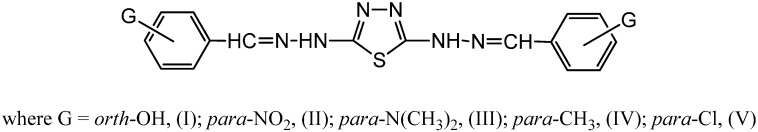
The synthesized Schiff bases.

The following compounds (Scheme 8) were all prepared by the method described previously [36].

### 3.2. Films Preparation

The polymer matrix used in this study was PVC (K value = 67, degree of polymerization = 800) supplied by Petkim (İzmir, Turkey). It was re-precipitated from THF solution by alcohol several times and finally dried under vacuum at room temperature for 24 h. PVC films were prepared as follows. The best solvent for PVC is THF. The films were prepared by dissolving 5 g of PVC in 100 g of THF under vigorous stirring for 30 min. It was necessary to control the hygrometry and the rate of evaporation of solvent during casting to maintain good optical quality and very limited turbidity. The film transmission should be greater than 80% in the near-UV range. After 3 h, the solution was spread on a slide stainless steel model (250, 120, 0.5 mm) and air-dried for 24 h. After the solvent evaporation, the samples were dried in a vacuum at room temperature for 30 h. The thickness of the resulting PVC film (30 µm) was measured by a micrometer Type 2610 A (Vogel Germany GmbH & Co. KG, Kevelaer, Germany).

### 3.3. Irradiation Experiments

Accelerated testing technique: The accelerated weatherometer QUV Accelerated Weathering Tester (Q. panel, Miami, FL, USA), was used for the irradiation of polymer films. The accelerated weathering tester contains a stainless steel plate, which has two holes on the front side and a third one behind. Each side contains a lamp (Fluorescent Ultraviolet Lights; 40 Watts). These lamps are of Type UV-B 313 (Q-Lab, Homestead, FL, USA) giving a spectrum range between 290 and 360 nm with a maximum wavelength at 313 nm. The polymer film samples were vertically fixed parallel to the lamps to make sure that the UV incident radiation is perpendicular to the samples. The irradiated samples are rotated from time to time to ensure that the intensity of the light incident on all samples is the same.

### 3.4. Photo-Degradation Measuring Methods

#### 3.4.1. Measuring the Photo-Degradation Rate of Polymer Films Using Infrared Spectrophotometry

The degree of photo-degradation of polymer film samples was followed by monitoring FTIR spectra in the range 4000 to 400 cm^−1^, using an spectra were obtained on a Nicolet 6700 FT-IR spectrophotometer (Thermo Nicolet Corp., Madison, WI, USA). The position of the carbonyl absorption is specified at 1722 cm^−1^, the polyene group at 1602 cm^−1^ and the hydroxyl group at 3500 cm^−1^ [28]. The progress of photo-degradation during different irradiation times was followed by observing the changes in the carbonyl and polyene peaks. Then, carbonyl (I_co_), polyene (I_po_) and hydroxyl (I_OH_) indices were calculated by comparison of the FTIR absorption peak at 1722, 1602 and 3500 cm^−1^ with the reference peak at 1328 cm^−1^, respectively. This method is called the band index method, which includes [8]: (All the equipment were supplied by Al-Nahrain University, Baghdad, Iraq).

The index of group can be determined according to Equation (3).

(3) $$\text{Is}=\frac{\text{As}}{\text{Ar}}\;$$

As = absorbance of peak under study; Ar = absorbance of reference peak; Is = index of group under study.

Actual absorbance, the difference between the absorbance of top peak and the baseline (top peak−baseline) is calculated using the baseline method [36].

#### 3.4.2. Determination of the Average Molecular Weight ${\text{(}\bar{\text{M}}}_{\text{v}}\text{)}$ Using the Viscometry Method

The viscosity property was used to determine the average molecular weight of the polymer, using the Mark–Houwink Equation (4) [37].

(4) $$\left[\eta \right]={\text{K}\bar{\text{M}}}_{\text{v}}^{\alpha }$$

[η] = the intrinsic viscosity; K and α are constants depending on the polymer-solvent system at a particular temperature.

The intrinsic viscosity of a polymer solution was measured with an Ostwald U-tube viscometer. Solutions were made by dissolving the polymer in a solvent (g/100 mL), and the flow times of the polymer solution and pure solvent are t and t_0_, respectively. Specific viscosity (η_sp_) was calculated as given in Equations (5) and (6):

(5) $$\eta_{\text{re}}=\frac{\text{t}}{{\text{t}}_{\text{o}}}\;$$

η_re_ = relative viscosity

η_sp_ = η_re_ − 1 (6)

The single point measurements were converted to intrinsic viscosities by Equation (7).

(7) [η]=(2/c) (ηsp−lnηre)12 

c = concentration of the polymer solution (g/100 mL).

By applying Equation (7), the molecular weight of degraded and virgin polymer can be calculated. Molecular weights of PVC with and without additives were calculated from intrinsic viscosities measured in THF solution using the following Equation (8):

(8) $$\left[\eta \right]=\text{4}.\text{17}\times {\text{10}}^{\text{-4}}{\text{Mv}}^{\text{0}.\text{6}}$$

The quantum yield of main chain scission (Φ_cs_)^11^ was calculated from viscosity measurement using the following Equation (9):

(9) Φcs=(CA/M¯v,o)[([ηo]/[η])1α−1]/Iot      

where C = concentration; A = Avogadro’s number; ${\text{(}\bar{\text{M}}}_{\text{v,o}}\text{)}$ = the initial viscosity average molecular weight; [η_o_] = intrinsic viscosity of PVC before irradiation; I_o_ = incident intensity; t = irradiation time in seconds.

## 4. Conclusions

In the work described in this paper, the photostabilization of polyvinyl chloride films using 2*N*-salicylidene-5-(substituted)-1,3,4-thiadiazole compounds was studied. These additives behave successfully as photostabilizers for PVC films. The additives take the following order in photostabilization activity according to their decrease in carbonyl, polyene and hydroxyl indices for PVC films: I > II > III > IV > V. These additives stabilize the PVC films through UV absorption or screening, peroxide decomposition and radical scavenging mechanisms. I was found to be the most efficient in the photostabilization process according to the photostability and mechanisms mentioned above. These mechanisms support the idea of using 2,5-dimercapto-1,3,4-thiadiazole compounds as commercial stabilizers for PVC.
